# Supplementary material for: Multidimensional coherent spectroscopy of correlated lattice systems
Source: NPJ Comput Mater. 2025 May 9;11(1):127. doi: 10.1038/s41524-025-01619-0 (PMC12064437; doi:10.1038/s41524-025-01619-0)
Supplement: Supplementary file 1 — SI_file [file 41524_2025_1619_MOESM1_ESM.pdf]

Multidimensional coherent spectroscopy of correlated lattice systems

SUPPLEMENTARY INFORMATION

A. High order Kubo formula

We consider a time dependent Hamiltonian in an interaction picture, where the full time dependent $\hat{H}(t)$ is split into a time independent part $\hat{H}_0$ and a time dependent perturbation $\hat{H}'$, i.e., $\hat{H}(t) = \hat{H}_0 + \hat{H}'(t)$. Defining $|\psi_I(t)\rangle = e^{i\hat{H}_0 t}|\psi_S(t)\rangle$ and $\hat{H}'_I(t) = e^{i\hat{H}_0 t}\hat{H}'(t)e^{-i\hat{H}_0 t}$, one obtains the Liouville-von Neumann equation of motion for the density matrix

$$\frac{\mathrm{d}}{\mathrm{d}t}\hat{\rho}_I(t) = -i[\hat{H}'_I(t), \hat{\rho}_I(t)]. \tag{1}$$

The solution is

$$\hat{\rho}_I(t_1) = \mathscr{U}_I^{\dagger}(t_1, t_0)\,\hat{\rho}_I(t_0)\mathscr{U}_I(t_1, t_0)\,,$$

with the time evolution operator $\mathscr{U}_I(t_1, t_0) = \mathcal{T}\exp\left[-i\int_{t_0}^{t} d\bar{t}\hat{H}'_I(\bar{t})\right]$. The density matrix $\hat{\rho}_I$ can be expanded in powers of $\hat{H}'$ as $\hat{\rho}_I(t) = \sum_i \hat{\rho}_I^{(i)}(t)$ [1], with

$$\hat{\rho}_I^{(i)}(t) = (-i)^n \int dt_i \int dt_{i-1} \ldots \int dt_1 [\hat{H}'_I(t_i), [\hat{H}'_I(t_{i-1}) \ldots, [\hat{H}'_I(t_1), \hat{\rho}(0)] \ldots]]. \tag{2}$$

For an operator $\hat{\boldsymbol{j}}$, the corresponding expectation value $\boldsymbol{j}(t)$ can also be expended into series $\hat{\boldsymbol{j}}(t) = \sum_i \hat{\boldsymbol{j}}^{(i)}(t)$, with

$$\boldsymbol{j}^{(i)}(t) = \mathrm{Tr}\left(\hat{\boldsymbol{j}}_I(t)\hat{\rho}^{(i)}(t)\right). \tag{3}$$

B. High order electric current in correlated lattice models

In the velocity gauge, the vector potential $\mathbf{A}(t)$ satisfies $\vec{\nabla}\cdot\mathbf{A} = 0$, representing a transverse electromagnetic field. The electrical current is obtained by $\hat{\boldsymbol{j}}(t) = -\delta\hat{H}(t)/\delta\boldsymbol{A}$ [2]. Specifically, for an interacting tight-binding model,

$$\begin{aligned} \hat{H}[\boldsymbol{A}(t)] &= -\sum_{i,j,s} h_{ij}\exp\left[ie\boldsymbol{A}(t)\cdot\boldsymbol{R}_{ij}\right]\hat{c}_{js}^{\dagger}\hat{c}_{is} + \hat{H}_{\mathrm{int}}, \\ \hat{\boldsymbol{j}}(t) &= -\frac{\delta\hat{H}}{\delta\boldsymbol{A}}(t) = ie\sum_{i,j,s} h_{ij}\boldsymbol{R}_{ij}\exp\left[ie\boldsymbol{A}(t)\cdot\boldsymbol{R}_{ij}\right]\hat{c}_{js}^{\dagger}\hat{c}_{is}. \end{aligned} \tag{4}$$

Combining the equations above, the n-th order current becomes

$$\boldsymbol{j}^{(i)}(t) = \mathrm{Tr}\left(\hat{\boldsymbol{j}}_I(t)\hat{\rho}^{(i)}(t)\right) = \mathrm{Tr}\left(\hat{\boldsymbol{j}}_I(t)(-i)^n\int dt_i\int dt_{i-1}\int dt_1[\hat{H}'_I(t_i), [\hat{H}'_I(t_{i-1}), [\ldots, [\hat{H}'_I(t_1), \hat{\rho}(0)]\ldots]]]\right). \tag{5}$$

The perturbation term

$$\hat{H}'_I(t) = e^{i\hat{H}_0 t}\left\{-\sum_{i,j,s} h_{ij}\left(1 - \exp\left[ie\boldsymbol{A}(t)\cdot\boldsymbol{R}_{ij}\right]\right)\hat{c}_{js}^{\dagger}\hat{c}_{is}\right)\right\}e^{-i\hat{H}_0 t}$$

vanishes when $\boldsymbol{A}(t) = 0$, and $\hat{H}_0$ is the Hamiltonian without laser field,

$$\hat{H}_0 = -\sum_{i,j,s} h_{ij}\hat{c}_{js}^{\dagger}\hat{c}_{is} + \hat{H}_{\mathrm{int}}. \tag{6}$$

Expanding $\hat{H}_I'(t)$ into a series of the vector potential $\mathbf{A}(t)$, we get [2]

$$\hat{H}_I'(t) = e^{i\hat{H}_0 t}\left\{-\hat{\boldsymbol{j}}_0\mathbf{A}(t) + \frac{1}{2}\hat{\tau}\mathbf{A}(t)\otimes\mathbf{A}(t) + \dots\right\}e^{-i\hat{H}_0 t}, \tag{7}$$

with the linear current operator $\hat{\boldsymbol{j}}_0$ and stress tensor operator $\hat{\tau}$ are given by

$$\hat{\boldsymbol{j}}_0 = ie\sum_{i,j,s} h_{ij}\mathbf{R}_{ij}c_{js}^\dagger c_{is}, \quad \hat{\tau} = e^2\sum_{i,j,s} h_{ij}\mathbf{R}_{ij}\otimes\mathbf{R}_{ij}c_{js}^\dagger c_{is}. \tag{8}$$

Finally, we can expand the current operator in the interaction picture into a series of $\mathbf{A}(t)$,

$$\hat{\boldsymbol{j}}_I(t) = -\frac{\delta\hat{H}_I}{\delta\boldsymbol{A}}(t) = e^{i\hat{H}_0 t}\left\{\hat{\boldsymbol{j}}_0 - \hat{\tau}\mathbf{A}(t) + \dots\right\}e^{-i\hat{H}_0 t}. \tag{9}$$

Now, we start from the zeroth order current in Eq. (5), where $\hat{\rho}^{(0)}(t) = \hat{\rho}(0)$ commutes with $e^{-i\hat{H}_0 t}$:

$$\boldsymbol{j}^{(0)}(t) = \mathrm{Tr}\left(\hat{\boldsymbol{j}}_I(t)\hat{\rho}^{(0)}(t)\right) = \langle\boldsymbol{j}_0\rangle - \langle\hat{\tau}\rangle\boldsymbol{A}(t) + \mathcal{O}(\mathbf{A}^2(t)). \tag{10}$$

Here, we have used the notation $\langle\dots\rangle \equiv \mathrm{Tr}(\dots\hat{\rho}(0))$. The first term vanishes when the system has no current before the perturbation. The second term vanishes when the vector potential is zero at time t. Next, for the first order current,

$$\begin{aligned}\boldsymbol{j}^{(1)}(t) &= \mathrm{Tr}\left(\hat{\boldsymbol{j}}_I(t)\hat{\rho}^{(1)}(t)\right) = \mathrm{Tr}\left(\hat{\boldsymbol{j}}_I(t)(-i)\int dt_1[\hat{H}_I'(t_1),\hat{\rho}(0)]\right)\\ &= -i\int_0^t dt_1\,\mathrm{Tr}\left(\hat{\boldsymbol{j}}_{0I}(t)\left[\hat{\boldsymbol{j}}_{0I}(t_1)\boldsymbol{A}(t_1),\hat{\rho}(0)\right]\right) + \mathcal{O}\left(\boldsymbol{A}(t)\boldsymbol{A}(t_1) + \boldsymbol{A}^2(t_1)\right)\\ &= -i\int_0^t dt_1\Big\langle\left[\hat{\boldsymbol{j}}_{0I}(t),\hat{\boldsymbol{j}}_{0I}(t_1)\right]\Big\rangle\boldsymbol{A}(t_1) + \mathcal{O}\left(\boldsymbol{A}(t)\boldsymbol{A}(t_1) + \boldsymbol{A}^2(t_1)\right).\end{aligned} \tag{11}$$

Within linear response theory, one defines the two-time optical conductivity

$$\sigma(t,t_1) \equiv \langle\tau\rangle_t - \int_0^t \chi(t,t_1)\,\mathrm{d}t_1 \quad (t > t_1 > 0) \tag{12}$$

with susceptibility

$$\chi(t,t_1) = i\Big\langle\left[\hat{\boldsymbol{j}}_{0I}(t),\hat{\boldsymbol{j}}_{0I}(t_1)\right]\Big\rangle. \tag{13}$$

Taking into account $\boldsymbol{A}(t_1) = -\int_0^{t_1}\boldsymbol{E}(\bar{t})\mathrm{d}\bar{t}$, the linear current $\boldsymbol{j}(t) = \int_0^t \mathrm{d}t_1\sigma(t,t_1)\boldsymbol{E}(t_1)$ is thus given by the zeroth and first order contribution above.

Similarly, for the second order current, we find

$$\begin{aligned}\boldsymbol{j}^{(2)}(t) &= \mathrm{Tr}\left(j_I(t)\hat{\rho}^{(2)}(t)\right) = \mathrm{Tr}\left(j_I(t)(-i)^2\int_0^{t_1}dt_2\int_0^t dt_1[\hat{H}_I'(t_2),[\hat{H}_I'(t_1),\hat{\rho}(0)]]\right)\\ &= (-i)^2\int_0^{t_1}dt_2\int_0^t dt_1\,\mathrm{Tr}\left(\hat{\boldsymbol{j}}_{0I}(t)\left[\hat{\boldsymbol{j}}_{0I}(t_2)\boldsymbol{A}(t_2),\left[\hat{\boldsymbol{j}}_{0I}(t_1)\boldsymbol{A}(t_1),\hat{\rho}(0)\right]\right]\right) + \mathcal{O}(\boldsymbol{A}^3)\\ &= (-i)^2\int_0^{t_1}dt_2\int_0^t dt_1\Big\langle\left[\hat{\boldsymbol{j}}_{0I}(t),\left[\hat{\boldsymbol{j}}_{0I}(t_2)\boldsymbol{A}(t_2),\hat{\boldsymbol{j}}_{0I}(t_1)\boldsymbol{A}(t_1)\right]\right]\Big\rangle + \mathcal{O}(\boldsymbol{A}^3),\end{aligned} \tag{14}$$

and for the third order current

$$\begin{aligned}\boldsymbol{j}^{(3)}(t) &= \mathrm{Tr}\left(\hat{\boldsymbol{j}}_I(t)\hat{\rho}^{(3)}(t)\right) = \mathrm{Tr}\left(\hat{\boldsymbol{j}}_I(t)(-i)^3\int_0^t dt_1\int_0^{t_1}dt_2\int_0^{t_2}dt_3[\hat{H}_I'(t_3),[\hat{H}_I'(t_2),[\hat{H}_I'(t_1),\hat{\rho}(0)]]]\right)\\ &= (-i)^3\int_0^t dt_1\int_0^{t_1}dt_2\int_0^{t_2}dt_3\,\mathrm{Tr}\Big\langle\left[\hat{\boldsymbol{j}}_{0I}(t),\left[\hat{\boldsymbol{j}}_{0I}(t_3)\boldsymbol{A}(t_3),\left[\hat{\boldsymbol{j}}_{0I}(t_2)\boldsymbol{A}(t_2),\hat{\boldsymbol{j}}_{0I}(t_1)\boldsymbol{A}(t_1)\right]\right]\right]\Big\rangle + \mathcal{O}(\boldsymbol{A}^4).\end{aligned} \tag{15}$$

C. Keldysh contour formalism (Liouville paths, double-sided Feymann diagram)

The Keldysh formalism provides a convenient framework for representing the nonequilibrium evolution of the density matrix. In the Schrödinger picture, the observable corresponding to the operator $\hat{O}$ is

$$O(t) = \mathrm{Tr}\left(\hat{\rho}(0)\hat{O}(t)\right) = \mathrm{Tr}\left[\mathscr{U}\left(t,t_0\right)\hat{\rho}(0)\mathscr{U}\left(t,t_0\right)^{\dagger}\hat{O}\right]$$

with $\mathscr{U}\left(t,t_0\right) = \mathcal{T}_t \exp[-i\int_{t_0}^{t} d\bar{t}\hat{H}(\bar{t})]$. In the Keldysh path integral formalism, one can separate the time dependent part $H'(t)$ from the time independent part $H_0(t)$,

$$\begin{aligned} O(t) &= \frac{1}{Z}\,\mathrm{Tr}\left[\mathcal{T}_{\mathcal{C}} e^{-i\int_{\mathcal{C}} d\bar{t}\hat{H}_0} e^{-i\int_{\mathcal{C}} d\bar{t}\hat{H}'(\bar{t})}\hat{O}\left(t_+\right)\right] \\ &= \frac{1}{Z}\,\mathrm{Tr}\left[\mathcal{T}_{\mathcal{C}} e^{-i\int_{\mathcal{C}} d\bar{t}\hat{H}_0} e^{-i\int_{\mathcal{C}_-} dt_-\hat{H}'(t_-)}\hat{O}\left(t_+\right) e^{-i\int_{\mathcal{C}_+} dt_+\hat{H}'(t_+)}\right] \\ &= \frac{1}{Z}\,\mathrm{Tr}\left[\mathcal{T}_{\mathcal{C}} e^{-i\int_{\mathcal{C}} d\bar{t}\hat{H}_0}\left(\sum_{n=0}^{\infty}(-i)^n\int_{\mathcal{C}^-} dt_n^- \ldots dt_1^-\hat{H}'(t_n^-)\ldots\hat{H}'(t_1^-)\right)\hat{O}\left(t^+\right)\right. \\ &\qquad\qquad \left.\left(\sum_{n=0}^{\infty}(-i)^n\int_{\mathcal{C}^+} dt_n^+ \ldots dt_1^+\hat{H}'(t_n^+)\ldots\hat{H}'(t_1^+)\right)\right]. \end{aligned} \tag{16}$$

Collecting the orders of H', one obtains the zero-th, first and second order electric current response,

$$\boldsymbol{j}^{(0)}(t) = \frac{1}{Z}\,\mathrm{Tr}\left[T_{\mathcal{C}} e^{-i\int_{\mathcal{C}} d\bar{t}\hat{H}_0(\bar{t})}\hat{\boldsymbol{j}}\left(t^+\right)\right], \tag{17}$$

$$\boldsymbol{j}^{(1)}(t) = \frac{(-i)}{Z}\,\mathrm{Tr}\left[T_{\mathcal{C}} e^{-i\int_{\mathcal{C}} d\bar{t}\hat{H}_0(\bar{t})}\left(\hat{\boldsymbol{j}}\left(t^+\right)\int_{\mathcal{C}^+} dt_1^+\hat{H}'(t_1^+) + \int_{\mathcal{C}^-} dt_1^-\hat{H}'(t_1^-)\hat{\boldsymbol{j}}\left(t^+\right),\right)\right] \tag{18}$$

$$\begin{aligned} \boldsymbol{j}^{(2)}(t) = \frac{(-i)^2}{Z}\,\mathrm{Tr}\Bigg[T_{\mathcal{C}} e^{-i\int_{\mathcal{C}} d\bar{t}\hat{H}_0(\bar{t})}\Bigg(&\hat{\boldsymbol{j}}\left(t^+\right)\iint_{\mathcal{C}^+} dt_2^+ t_1^+\hat{H}'(t_2^+)\hat{H}'(t_1^+) + \int_{\mathcal{C}^+} dt_2^+\int_{\mathcal{C}^-} dt_1^-\hat{H}'(t_2^-)\hat{\boldsymbol{j}}\left(t^+\right)\hat{H}'(t_1^-) \\ &+ \int_{\mathcal{C}^+} dt_1^+\int_{\mathcal{C}^-} dt_2^-\hat{H}'(t_1^-)\hat{\boldsymbol{j}}\left(t^+\right)\hat{H}'(t_2^-) + \iint_{\mathcal{C}^-} dt_2^- dt_1^-\hat{H}'(t_2^-)\hat{H}'(t_1^-)\hat{\boldsymbol{j}}\left(t^+\right)\Bigg)\Bigg]. \end{aligned} \tag{19}$$

We are interested in the third order current $\boldsymbol{j}^{(3)}(t)$. Expanding $\hat{H}'$ in powers of $\boldsymbol{A}$ in the weak field limit gives $\hat{H}'(t) = \hat{\boldsymbol{j}}(t)\boldsymbol{A}(t) + \mathcal{O}(\mathbf{A}^2)$. The third order current thus includes eight terms, which we can write as

$$\boldsymbol{j}^{(3)}(t) = \frac{(-i)^3}{Z}\,\mathrm{Tr}\left[T_{\mathcal{C}} e^{-i\int_{\mathcal{C}} d\bar{t}\hat{H}_0(\bar{t})}\iiint_{\mathcal{C}_{+/-}} dt_3 dt_2 dt_1 \boldsymbol{A}(t_3)\boldsymbol{A}(t_2)\boldsymbol{A}(t_1)R^{(3)}(t,t_3,t_2,t_1)\right] + \mathcal{O}(A^4), \tag{20}$$

with the four point response "generator" ($t > t_3 > t_2 > t_1$)

$$\begin{aligned} R^{(3)}(t,t_3,t_2,t_1) =& \hat{\boldsymbol{j}}_{1-}\hat{\boldsymbol{j}}_{3-}\hat{\boldsymbol{j}}_{t}\hat{\boldsymbol{j}}_{2+} + \hat{\boldsymbol{j}}_{2-}\hat{\boldsymbol{j}}_{t}\hat{\boldsymbol{j}}_{3+}\hat{\boldsymbol{j}}_{1+} \\ & \hat{\boldsymbol{j}}_{1-}\hat{\boldsymbol{j}}_{2-}\hat{\boldsymbol{j}}_{t}\hat{\boldsymbol{j}}_{3+} + \hat{\boldsymbol{j}}_{3-}\hat{\boldsymbol{j}}_{t}\hat{\boldsymbol{j}}_{2+}\hat{\boldsymbol{j}}_{1+} \\ & \hat{\boldsymbol{j}}_{2-}\hat{\boldsymbol{j}}_{3-}\hat{\boldsymbol{j}}_{t}\hat{\boldsymbol{j}}_{1+} + \hat{\boldsymbol{j}}_{1-}\hat{\boldsymbol{j}}_{t}\hat{\boldsymbol{j}}_{3+}\hat{\boldsymbol{j}}_{2+} \\ & \hat{\boldsymbol{j}}_{t}\hat{\boldsymbol{j}}_{3+}\hat{\boldsymbol{j}}_{2+}\hat{\boldsymbol{j}}_{1+} + \hat{\boldsymbol{j}}_{1-}\hat{\boldsymbol{j}}_{2-}\hat{\boldsymbol{j}}_{3-}\hat{\boldsymbol{j}}_{t}. \end{aligned} \tag{21}$$

Here, $\hat{\boldsymbol{j}}_{i\pm}$ is a short hand notation for $\hat{\boldsymbol{j}}(t_i)$, $t_i \in \mathcal{C}_{\pm}$. The Keldysh index of t is dropped due to the fact that $\hat{\boldsymbol{j}}(t^+) = \hat{\boldsymbol{j}}(t^-)$ at the end of the real time branches of the Keldysh contour. The time integration on the $\mathcal{C}_-$ branch brings a negative sign compared to a normal time integral.

For simplicity, we consider the semi-impulsive limit, i.e., assume an external field $\boldsymbol{A}(t) \propto \delta(t)\cos(\omega t - \boldsymbol{k}\cdot\boldsymbol{r}) = \delta(t)\left(e^{-i\omega t + i\boldsymbol{k}\cdot\boldsymbol{r}} + e^{i\omega t - i\boldsymbol{k}\cdot\boldsymbol{r}}\right)\hat{\boldsymbol{r}}$ which is the sum of an excitation and deexcitation. If a given pulse would create additional excitations and deexcitations along the contour, it would complicate the analysis.

In the non-collinear setup, we can selectively detect the signal in a given direction. For example, in the "box" geometry of four-wave-mixing, after three non-collinear pump pulses, we can put the fourth pump (local oscillator) along the direction $\vec{k}_{\text{sig}} = (-\vec{k}_A + \vec{k}_B + \vec{k}_C)$. By ordering the time sequences of the laser pulses A, B, C, we can select the excitation or deexcitation term of the light-matter interaction.

Following the notations in Ref. 3, if k_A comes first, the t_1 pulse deexcites, while t_2, t_3 excites along the contour. Three of the pathways survive, the rephasing diagrams R_1, R_2, and R_3 (top panels of Fig. 1**b**). If k_B comes first, the t_2 pulse excites while t_2, t_3 deexcites, as illustrated by the nonrephasing diagrams R_4, R_5, R_6 in the middle panels of Fig. 1**b**. If k_C comes first, t_3 excites and t_1, t_2 deexcites, as in the two 2Q pathways R_7, R_8 in the bottom panels of Fig. 1**b**.

We note that in the presence of inhomogeneous broadening of the excitations, the R signals (R_1 ,R_2, R_3) rephase when $t = \tau$, while the NR signals (R_4 ,R_5, R_6) keep dephasing. Consider an excitation energy centered at ω_0 with Gaussian broadening of width $\Delta\omega$. Neglecting the decoherence during the time evolution, the R and NR signals are $S^{\text{R/NR}}(\tau, t)$. The R signal echos when $t = \tau$, since the factor $e^{-(\tau-t)^2\Delta\omega^2/2} = 1$, while the NR signal with $e^{-(\tau+t)^2\Delta\omega^2/2}$ keeps decreasing.

For comparison, we also plot the traditional double-sided Feymann diagrams in Fig. 1**c**. In Table I, the signal locations of each pathway in the (ω_τ, ω_t) domains are listed, together with the frequencies of the intensity oscillations during T.

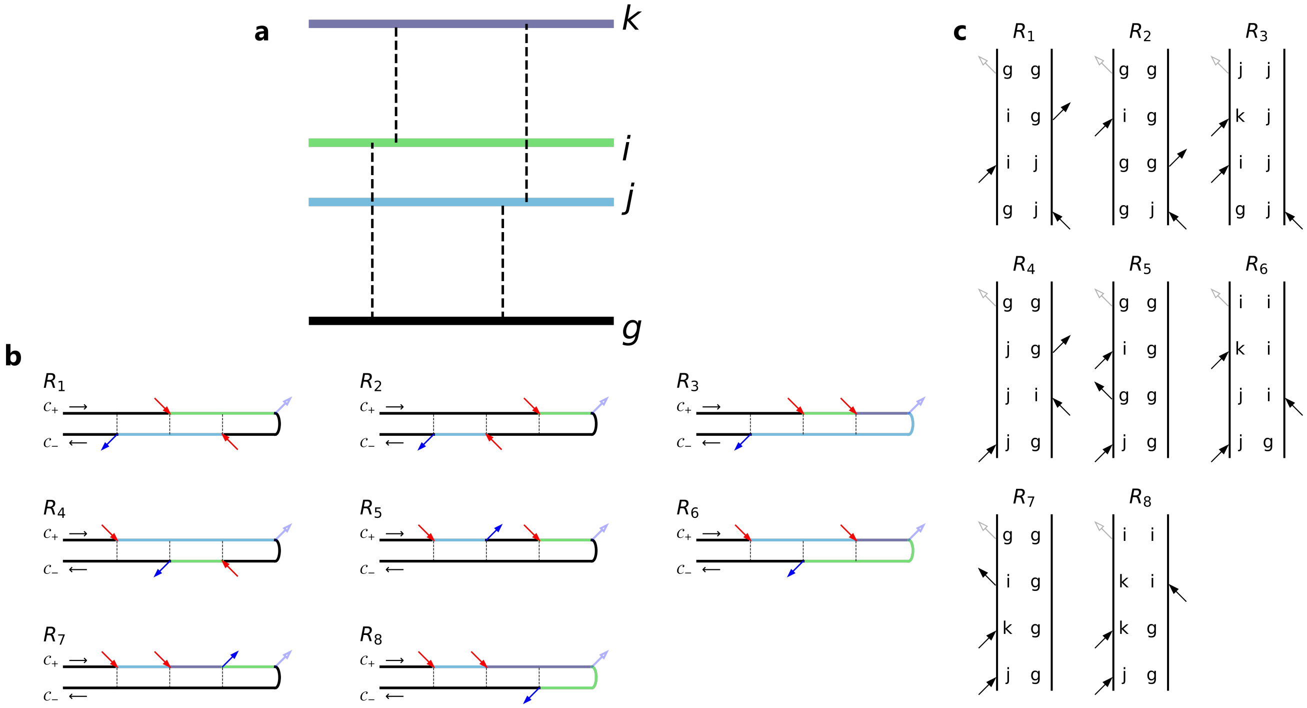

Figure 1. **a**, Energy levels of a generic quantum system. **b**, Keldysh contours for the R (top), NR (middle) and 2Q (bottom) signals R_1-R_8. **c**, Double-sided Feymann diagrams for the same signals as in **b**.

[1] Nozières, P. *Theory of interacting Fermi systems*. Advanced book classics (Perseus Publishing, Cambridge, Mass, 1997).

[2] Lenarčič, Z., Golež, D., Bonča, J. & Prelovšek, P. Optical response of highly excited particles in a strongly correlated system. *Physical Review B* **89**, 125123 (2014).

[3] Hamm, P. & Zanni, M. *Concepts and Methods of 2D Infrared Spectroscopy* (Cambridge University Press, Cambridge, 2011).

	Signal index	Peak location (ω_τ,ω_t)	Oscillation frequency Ω
R	R_1	$(-\omega_j, \omega_i)$	ω_{ij}
	R_2	$(-\omega_j, \omega_i)$	ω_{ij}
	R_3	$(-\omega_j, \omega_{jk})$	ω_{ij}
NR	R_4	(ω_j, ω_j)	ω_{ij}
	R_5	(ω_j, ω_i)	0
	R_6	(ω_j, ω_{ik})	ω_{ij}
2Q	R_7	(ω_j, ω_i)	ω_k
	R_8	(ω_j, ω_{ik})	ω_k

Table I. Locations of the R (top), NR (middle) and 2Q (bottom) signals in the (ω_τ, ω_t) plane and oscillation frequencies of the signal intensities during the waiting time T.
